# Development and internal validation of a prediction model for hypoxic hepatitis after coronary artery bypass grafting with cardiopulmonary bypass

**DOI:** 10.3389/fmed.2026.1785046

**Published:** 2026-06-08

**Authors:** Yan Cui, Ya Mei Sun, Jie Zhang

**Affiliations:** Department of Gastroenterology, Beijing Anzhen Hospital, Capital Medical University, Beijing, China

**Keywords:** cardiopulmonary bypass, coronary artery bypass grafting, hypoxic hepatitis, ischemic hepatitis, nomogram

## Abstract

**Background:**

Hypoxic hepatitis (HH) after on-pump coronary artery bypass grafting (CABG) is associated with poor outcomes, but dedicated prediction tools are lacking. We developed and internally validated a parsimonious model for severe postoperative aminotransferase elevation compatible with HH.

**Methods:**

This retrospective single-center study included 626 consecutive adults who underwent CABG with cardiopulmonary bypass at Beijing Anzhen Hospital between June 2020 and June 2023. The outcome was peak postoperative alanine aminotransferase or aspartate aminotransferase ≥800 U/L after exclusion of alternative causes of acute liver injury during data extraction. Missing predictor data were addressed with random-forest multiple imputation (20 datasets). Prespecified predictors were screened with LASSO penalized logistic regression in each imputed dataset using 10-fold cross-validation; stably selected variables entered the final logistic model. Internal validation used bootstrap optimism correction, with temporal split and complete-case analyses as secondary analyses.

**Results:**

The outcome occurred in 57/626 patients (9.1%) and was associated with higher in-hospital mortality (49.1% vs. 4.6%; *P* < 0.001). Final predictors were total sternotomy count (OR 3.533, 95% CI 1.724–7.241), cardiopulmonary bypass duration (OR 1.047 per minute, 95% CI 1.035–1.059), and peak intraoperative lactate (OR 1.403 per mmol/L, 95% CI 1.251–1.574). Apparent and optimism-corrected AUCs were 0.907 and 0.903; temporal validation AUC was 0.955.

**Conclusion:**

A three-predictor model estimated the risk of severe postoperative aminotransferase elevation compatible with HH after on-pump CABG and may support intraoperative or early postoperative risk awareness. External validation is required before routine use.

## Introduction

Hypoxic hepatitis (HH), also referred to as ischemic hepatitis or shock liver, is classically characterized by a sudden, marked, and usually transient rise in aminotransferases in the setting of cardiac, circulatory, or respiratory failure after exclusion of alternative hepatotoxic causes. Contemporary reviews commonly use an aminotransferase threshold of at least 20 times the upper limit of normal as a pragmatic marker of severe hepatocellular injury, although specific diagnostic frameworks vary somewhat across studies (1–4). In most clinical descriptions, HH is recognized not only by profound biochemical injury but also by its occurrence in a relevant hypoxic or hemodynamic context and by careful exclusion of competing etiologies such as viral hepatitis, biliary obstruction, or drug-induced liver injury (1–4). In intensive care unit cohorts, HH is consistently associated with high short-term mortality and frequent multiorgan dysfunction, underscoring that it is both a hepatic complication and a marker of severe systemic derangement (5, 6).

Mechanistically, HH reflects the interaction of reduced hepatic arterial and portal venous inflow, venous congestion related to right-sided cardiac dysfunction, and systemic arterial hypoxemia. These insults can result in centrilobular hypoxic hepatocellular injury and may be further amplified by reperfusion-related inflammation after restoration of systemic perfusion. Recent critical care literature has reinforced the prognostic relevance of severe aminotransferase elevation in global hypoperfusion states. In post-cardiac-arrest populations, for example, HH has been associated with substantially worse survival and neurological outcomes, as well as higher lactate concentrations, greater vasopressor requirements, and more frequent renal replacement therapy, supporting the view that HH tracks with profound circulatory and metabolic stress rather than isolated liver injury (1, 4, 7, 8).

After cardiac surgery, severe postoperative liver injury may be precipitated by low cardiac output, prolonged cardiopulmonary bypass (CPB) exposure, perioperative arrhythmia, hypoxemia, venous congestion, overt shock, or combinations of these insults. CABG performed with CPB may be particularly relevant in this regard because the perioperative course can include non-pulsatile flow, hemodilution, ischemia-reperfusion phenomena, systemic inflammatory activation, and transient impairment of hepatic oxygen delivery. Prior observational studies have emphasized both the epidemiologic burden and the adverse prognosis of HH in critically ill populations, and work in specific cardiac surgical settings has suggested that factors such as longer CPB duration and poorer baseline hepatic reserve may increase postoperative risk (6, 7). Nevertheless, dedicated perioperative risk-estimation tools for postoperative HH remain limited, and the few available prediction studies have mainly focused on selected aortic procedures or broader postoperative liver injury rather than CABG performed with CPB (9).

This gap is clinically important because postoperative HH-compatible liver injury is uncommon but potentially devastating, and earlier recognition may support more vigilant surveillance for systemic hypoperfusion, closer postoperative liver-biochemistry monitoring, and intensified supportive management in high-risk patients. A parsimonious model based on routinely available perioperative variables may therefore be more practical than complex tools requiring specialized measurements. Accordingly, the present study aimed to determine the incidence and clinical correlates of severe postoperative aminotransferase elevation compatible with HH after CPB-assisted CABG, to identify key perioperative predictors, and to develop and internally validate a clinically usable prediction model using contemporary regression-based and exploratory machine-learning approaches.

## Materials and methods

### Patients and data collection

We performed a retrospective analysis of consecutive adult patients with coronary artery disease who underwent CABG with CPB at Beijing Anzhen Hospital, Capital Medical University, between June 9, 2020 and June 14, 2023. A total of 626 patients were available in the revised full-cohort analysis. In the original data-cleaning workflow, 26 patients with incomplete clinical data required for candidate-predictor assessment were excluded from the complete-case dataset, leaving 600 patients; however, in the revised primary analysis these partially incomplete cases were retained and handled using multiple imputation. The study protocol was reviewed and approved by the Institutional Ethics Committee of Beijing Anzhen Hospital (Approval No. 2025351X). The requirement for written informed consent was waived because of the retrospective design and use of anonymized patient data.

Baseline demographics, perioperative characteristics, laboratory data, and in-hospital outcomes were extracted from electronic medical records, anesthesia and perfusion records, operative notes, and laboratory systems. Collected variables included age, sex, height, weight, body mass index, smoking and alcohol history, hypertension, diabetes mellitus, hyperlipidemia, lung disease, New York Heart Association class, old myocardial infarction, left main coronary artery disease, peripheral arterial disease, total sternotomy count, number of grafts performed, CPB duration, peak intraoperative lactate, preoperative left ventricular ejection fraction, preoperative ALT, AST, albumin, alkaline phosphatase, gamma-glutamyl transferase, total bilirubin, international normalized ratio, postoperative peak ALT and AST, ICU stay, total hospital stay, and in-hospital all-cause mortality. Yan Cui and YaMei Sun performed data collection and data checking according to predefined variable definitions. When discrepancies in extracted values, eligibility, or exclusion of alternative hepatic causes occurred, Jie Zhang made the final decision after review of the available source records.

### Outcome definition

The primary outcome was the occurrence of severe postoperative aminotransferase elevation compatible with HH after CABG with CPB. For reproducibility in this retrospective study, the outcome was defined operationally as peak postoperative alanine aminotransferase (ALT) or aspartate aminotransferase (AST) ≥ 800 U/L during the index hospitalization after CABG with CPB, corresponding to at least 20 times the upper limit of normal when 40 U/L is used as the reference upper limit. This threshold is consistent with the severe aminotransferase elevations commonly used in contemporary HH and ischemic liver injury literature (1, 2, 4, 10–16). During data extraction and review of source records, patients with clear alternative explanations for acute liver injury, including viral hepatitis, biliary obstruction, drug-induced liver injury, or known severe pre-existing liver disease, were excluded from outcome classification in accordance with standard HH diagnostic principles (1, 2, 4, 11, 14, 17).

Because the present dataset was retrospective, a prospective blinded adjudication committee and formal inter-rater reliability assessment were not available. In addition, detailed hemodynamic descriptors such as sustained hypotension, shock, low cardiac output, mechanical circulatory support, cardiac arrest, and severe respiratory failure were not uniformly available in structured form for every patient. Therefore, the outcome should be interpreted as an operational endpoint of severe postoperative aminotransferase elevation compatible with HH rather than as a prospectively adjudicated HH diagnosis. Lactate level and CPB duration were not used as formal components of the biochemical outcome threshold or the alternative-cause exclusion rule, but were evaluated as candidate predictors.

### Candidate predictors

Candidate predictors were prespecified before model fitting on the basis of clinical plausibility, routine perioperative availability, and prior literature on HH and postoperative hypoperfusion-related organ injury (6, 7, 15, 18–22). The prespecified candidate set comprised age, sex, smoking and alcohol history, hypertension, diabetes mellitus, hyperlipidemia, lung disease, New York Heart Association class, old myocardial infarction, left main coronary artery disease, peripheral arterial disease, total sternotomy count, number of grafts, CPB duration, body mass index, preoperative left ventricular ejection fraction, preoperative ALT, AST, albumin, alkaline phosphatase, gamma-glutamyl transferase, total bilirubin, international normalized ratio, and peak intraoperative lactate. Postoperative peak ALT/AST, ICU stay, total hospital stay, and in-hospital death were not considered candidate predictors because they are outcomes or downstream consequences rather than predictors available at the intended time of use. Intraoperative adverse events were extracted for descriptive analyses, but were not forced into the revised predictor set used for penalized regression screening and final model development.

### Missing data and multiple imputation

Missingness was low but was handled as part of the primary analysis rather than by casewise deletion. Missing predictor data were imputed using multiple imputation by chained equations with random-forest imputation, creating 20 imputed datasets with 20 iterations (23, 24). The outcome was not imputed. Regression estimates from analyses across the completed datasets were pooled using Rubin‘s rules.

### Feature selection and primary model

Given the limited event count, feature selection used LASSO penalized logistic regression alone to support a parsimonious and reproducible model-building strategy (25, 26). Rather than relying on a single completed dataset, LASSO was run separately in each of the 20 imputed datasets. Ten-fold cross-validation was performed within each imputed dataset, and the more conservative lambda.1se solution was used as the primary criterion to assess selection stability across imputations; lambda.min was examined as a supportive, less conservative reference. Variables showing stable selection across imputations and retaining clinical plausibility were carried forward into the final standard logistic regression model, and final regression estimates were pooled across imputations using Rubin’s rules. The final model included total sternotomy count, CPB duration, and peak intraoperative lactate.

**FIGURE 1:**
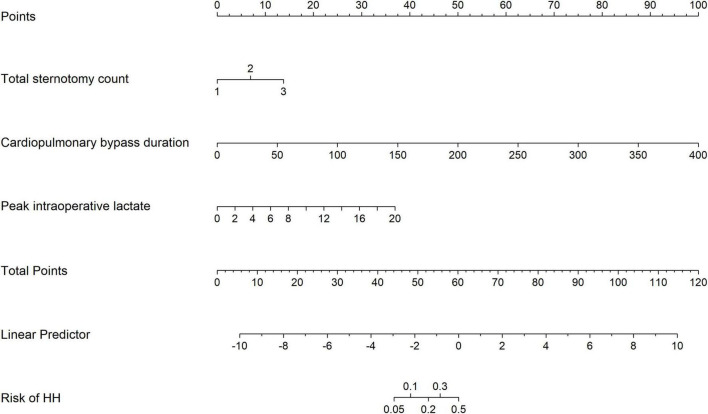
Nomogram based on total sternotomy count, CPB duration, and peak intraoperative lactate.

Categorical variables were summarized as counts and percentages. Continuous variables were summarized as medians with interquartile ranges or means with standard deviations, as appropriate to their observed distributions. Group comparisons were performed using the chi-square test or Fisher’s exact test for categorical variables and the Student’s *t*-test or Wilcoxon rank-sum test for continuous variables, as appropriate. The primary logistic regression model was fitted in the full cohort to maximize statistical efficiency. A nomogram (Figure 1) based on the final logistic regression model was constructed for visual risk estimation (26). Model discrimination was assessed by the area under the receiver operating characteristic curve (AUC) with 95% CI. Overall accuracy was assessed using the Brier score. Calibration was summarized using calibration intercept and calibration slope and visualized with bootstrap-corrected calibration plots. Clinical utility was explored using decision curve analysis. A Youden-index probability cutoff was calculated only for exploratory description and was not considered a recommended clinical decision threshold.

### Internal validation and sensitivity analyses

Internal validation was performed using 1,000 bootstrap resamples to estimate optimism-corrected AUC, Brier score, calibration intercept, and calibration slope, in line with modern regression-model validation principles (26). A temporal split analysis was retained as a secondary internal validation sensitivity analysis. Patients admitted from June 9, 2020 to July 28, 2022 formed the temporal training cohort, and those admitted from August 2, 2022 to June 14, 2023 formed the temporal validation cohort. In addition, a complete-case sensitivity analysis was performed in the original 600-patient cohort, and the same three-predictor logistic model was refitted in that cohort.

### Exploratory machine-learning analyses

Random forest and XGBoost models were fitted in the 626-patient cohort using the same three predictors as the logistic model. These analyses were explicitly considered exploratory because the small number of predictors limits the advantage of flexible machine-learning algorithms, and the event count is modest. Models were evaluated using repeated 5-fold cross-validation with 5 repeats. Apparent performance was also reported to identify overfitting but was not used to claim model superiority. The random-forest component of this exploratory comparison was informed by classical ensemble-learning principles (27).

### Reporting

The study was reported with reference to TRIPOD + AI (28). Risk of bias and applicability were assessed using the PROBAST + AI framework (29). Statistical analyses were performed using R version 4.4.2. A two-sided *P* < 0.05 was considered statistically significant.

## Results

### Study cohort, missing data, and clinical characteristics

The analysis included 626 patients, of whom 57 (9.1%) developed severe postoperative aminotransferase elevation compatible with HH and 569 (90.9%) did not. Missing data were infrequent overall. The pattern of missing predictor data is summarized in Supplementary Table 1, and descriptive characteristics in the first completed imputed dataset are shown in Supplementary Table 2. The variables with the highest proportions of missingness were NYHA class (8/626, 1.28%), height (7/626, 1.12%), INR (6/626, 0.96%), ALP (3/626, 0.48%), GGT (3/626, 0.48%), and preoperative LVEF (3/626, 0.48%). No missing values were present for the outcome, CPB duration, peak intraoperative lactate, or total sternotomy count. Baseline characteristics, operative variables, and postoperative outcomes are summarized in Table 1.

**TABLE 1:** Baseline characteristics, preoperative laboratory indices, operative variables, and postoperative outcomes of 626 patients undergoing CABG with CPB.

Variable	Overall (*n* = 626)	Non-HH (*n* = 569)	HH (*n* = 57)	*P*-value
Demography and baseline history				
*n*	626	569	57	
Age, years, median [IQR]	63.00 [56.00, 68.00]	62.00 [56.00, 68.00]	65.00 [58.00, 70.00]	0.221
Height, m, median [IQR]	1.68 [1.61, 1.72]	1.68 [1.62, 1.72]	1.65 [1.60, 1.72]	0.254
Weight, kg, median [IQR]	71.00 [63.00, 78.00]	71.00 [63.00, 79.00]	68.00 [65.00, 77.00]	0.267
BMI, kg/m^2^, median [IQR]	25.40 [23.39, 27.50]	25.38 [23.42, 27.55]	25.53 [22.86, 27.06]	0.823
Female sex, *n* (%)	140 (22.4)	125 (22.0)	15 (26.3)	0.559
Smoking history, *n* (%)	305 (48.7)	273 (48.0)	32 (56.1)	0.3
Alcohol use, *n* (%)	172 (27.5)	157 (27.6)	15 (26.3)	0.96
Medical history and operative variables				
Total sternotomy count, median [IQR]	1.00 [1.00, 1.00]	1.00 [1.00, 1.00]	1.00 [1.00, 2.00]	<0.001
Hypertension, *n* (%)	416 (66.5)	376 (66.1)	40 (70.2)	0.633
Diabetes mellitus, *n* (%)	266 (42.5)	239 (42.0)	27 (47.4)	0.522
Hyperlipidemia, *n* (%)	353 (56.4)	314 (55.2)	39 (68.4)	0.075
Lung disease, *n* (%)	79 (12.6)	72 (12.7)	7 (12.3)	1
NYHA class ≥ III, *n* (%)	306 (48.9)	272 (47.8)	34 (59.6)	0.117
Old myocardial infarction, *n* (%)	317 (50.6)	287 (50.4)	30 (52.6)	0.86
Left main disease, *n* (%)	131 (20.9)	118 (20.7)	13 (22.8)	0.845
Peripheral arterial disease, *n* (%)	310 (49.5)	270 (47.5)	40 (70.2)	0.002
Number of grafts, median [IQR]	3.00 [3.00, 4.00]	3.00 [3.00, 4.00]	3.00 [3.00, 4.00]	0.719
Intraoperative adverse events, *n* (%)	130 (20.8)	109 (19.2)	21 (36.8)	0.003
Peak intraoperative lactate, mmol/L, median [IQR]	3.75 [2.40, 6.71]	3.30 [2.25, 6.15]	7.07 [5.80, 9.93]	<0.001
CPB duration, min, median [IQR]	116.50 [95.00, 142.75]	113.00 [91.00, 138.00]	171.00 [137.00, 182.00]	<0.001
Preoperative laboratory and cardiac indices				
Preoperative LVEF, %, median [IQR]	58.00 [50.00, 63.00]	58.00 [50.00, 63.00]	55.00 [42.00, 61.00]	0.151
Preoperative ALT, U/L, median [IQR]	21.00 [14.00, 31.00]	21.00 [14.00, 31.00]	20.00 [14.00, 29.00]	0.613
Preoperative AST, U/L, median [IQR]	18.00 [15.00, 24.75]	18.00 [15.00, 24.00]	19.00 [15.00, 27.00]	0.478
Preoperative albumin, g/L, mean (SD)	42.69 (3.67)	42.72 (3.65)	42.34 (3.92)	0.46
Preoperative ALP, U/L, median [IQR]	75.00 [63.00, 89.00]	75.00 [63.00, 88.00]	73.00 [63.00, 93.00]	0.958
Preoperative GGT, U/L, median [IQR]	26.50 [19.00, 40.00]	26.00 [19.00, 40.00]	28.00 [18.00, 44.00]	0.794
Preoperative TBIL, μmol/L, median [IQR]	10.44 [7.70, 14.00]	10.51 [7.73, 14.10]	9.74 [7.05, 13.63]	0.553
Preoperative INR, median [IQR]	1.01 [0.97, 1.05]	1.01 [0.97, 1.05]	1.01 [0.97, 1.05]	0.841
Postoperative outcomes				
Peak postoperative ALT, U/L, median [IQR]	50.50 [26.25, 111.75]	45.00 [25.00, 86.00]	1802.10 [895.00, 3100.00]	<0.001
Peak postoperative AST, U/L, median [IQR]	61.00 [37.00, 118.00]	56.00 [36.00, 95.00]	2939.00 [1541.00, 6381.00]	<0.001
Hospital length of stay, days, median [IQR]	15.00 [11.00, 19.00]	15.00 [11.00, 19.00]	18.00 [12.00, 24.00]	0.005
ICU length of stay, days, median [IQR]	3.00 [2.00, 5.00]	3.00 [2.00, 4.00]	8.00 [4.00, 11.00]	<0.001
In-hospital mortality, *n* (%)	54 (8.6)	26 (4.6)	28 (49.1)	<0.001

HH, hypoxic hepatitis; CABG, coronary artery bypass grafting; CPB, cardiopulmonary bypass; BMI, body mass index; NYHA, New York Heart Association; LVEF, left ventricular ejection fraction; ALT, alanine aminotransferase; AST, aspartate aminotransferase; ICU, intensive care unit.

The median age of the overall cohort was 63 years [56–68], the median body mass index was 25.40 kg/m2 [23.39–27.50], and 22.4% of patients were female. Hypertension, diabetes mellitus, and hyperlipidemia were present in 66.5, 42.5, and 56.4% of patients, respectively, and 48.9% had NYHA class >≥ III. Baseline demographics were broadly similar between patients with and without the outcome, including age (65 [58–70] vs. 62 [56–68] years, *P* = 0.221), body mass index (25.53 [22.86–27.06] vs. 25.38 [23.42–27.55] kg/m^2^, *P* = 0.823), and female sex (26.3% vs. 22.0%, *P* = 0.559).

By contrast, several perioperative characteristics differed materially between groups. Patients with the outcome had a higher total sternotomy count (1.00 [1.00–2.00] vs. 1.00 [1.00–1.00], *P* < 0.001), a higher prevalence of peripheral arterial disease (70.2% vs. 47.5%, *P* = 0.002), and more frequent intraoperative adverse events (36.8% vs. 19.2%, *P* = 0.003). They also had substantially longer CPB duration (171 [137–182] vs. 113 [91–138] minutes, *P* < 0.001) and higher peak intraoperative lactate (7.07 [5.80–9.93] vs. 3.30 [2.25–6.15] mmol/L, *P* < 0.001). Preoperative LVEF was numerically lower in the outcome group, but the difference did not reach statistical significance (55 [42–61] vs. 58 [50–63], *P* = 0.151).

Postoperative biochemical and clinical outcomes were markedly worse among patients with the outcome. Peak postoperative ALT was 1802.10 [895.00–3100.00] U/L vs. 45.00 [25.00–86.00] U/L, and peak postoperative AST was 2939.00 [1541.00–6381.00] U/L vs. 56.00 [36.00–95.00] U/L (both *P* < 0.001). ICU stay (8 [4–11] vs. 3 [2–4] days, *P* < 0.001), total hospital stay (18 [12–24] vs. 15 [11–19] days, *P* = 0.005), and in-hospital mortality (49.1% vs. 4.6%, *P* < 0.001) were also substantially higher in the outcome group.

### Penalized regression variable selection

In LASSO penalized logistic regression across 20 imputed datasets, CPB duration and peak intraoperative lactate were selected in 20 of 20 datasets using the conservative lambda.1se criterion. Total sternotomy count was selected in 15 of 20 datasets by lambda.1se and in 20 of 20 datasets by lambda.min. Peripheral arterial disease and hyperlipidemia were selected only intermittently by lambda.min and were not retained under lambda.1se. We did not use the first completed dataset alone for model selection. Instead, variable-selection stability was summarized across all imputations, and total sternotomy count was retained in the final model on the basis of moderate-to-high selection stability together with clinical plausibility. Detailed LASSO selection stability across imputations is provided in Supplementary Table 3.

### Primary logistic regression model

In the pooled multiple-imputation analysis, all three final predictors were independently associated with the outcome. Each additional sternotomy was associated with higher odds of the outcome (OR 3.533, 95% CI 1.724–7.241, *P* < 0.001). CPB duration was associated with higher odds per minute (OR 1.047, 95% CI 1.035–1.059, *P* < 0.001), and peak intraoperative lactate was associated with higher odds per mmol/L (OR 1.403, 95% CI 1.251–1.574, *P* < 0.001). The pooled multivariable logistic regression results are summarized in Table 2. Detailed pooled regression estimates and multicollinearity diagnostics are provided in Supplementary Tables 4,5.

**TABLE 2:** Pooled multivariable logistic regression results after multiple imputation.

Predictor	OR	Lower 95% CI	Upper 95% CI	*P*-value
Total sternotomy count	3.533	1.724	7.241	<0.001
CPB duration, per minute	1.047	1.035	1.059	<0.001
Peak intraoperative lactate, per mmol/L	1.403	1.251	1.574	<0.001

### Model performance and internal validation

**FIGURE 2:**
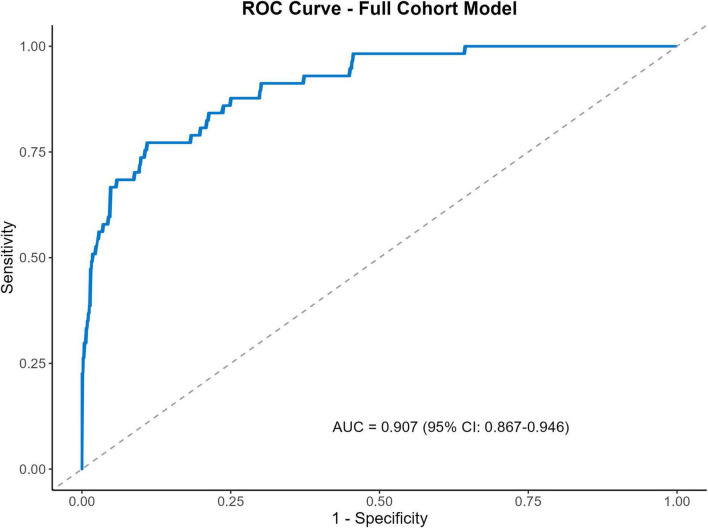
Receiver operating characteristic curve for the full-cohort logistic model.

**FIGURE 3:**
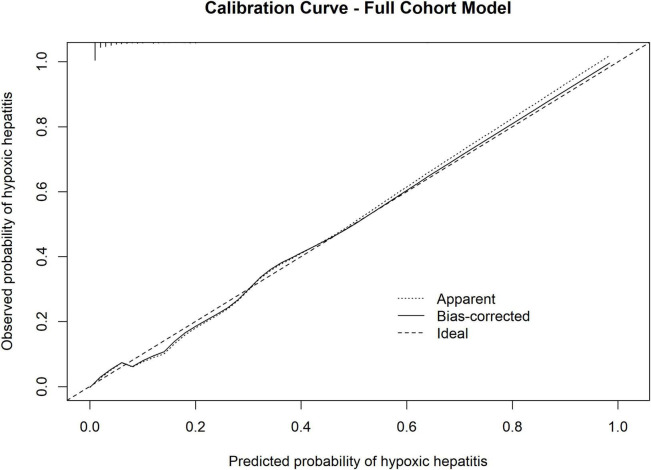
Bootstrap-corrected calibration curve for the full-cohort logistic model.

**FIGURE 4:**
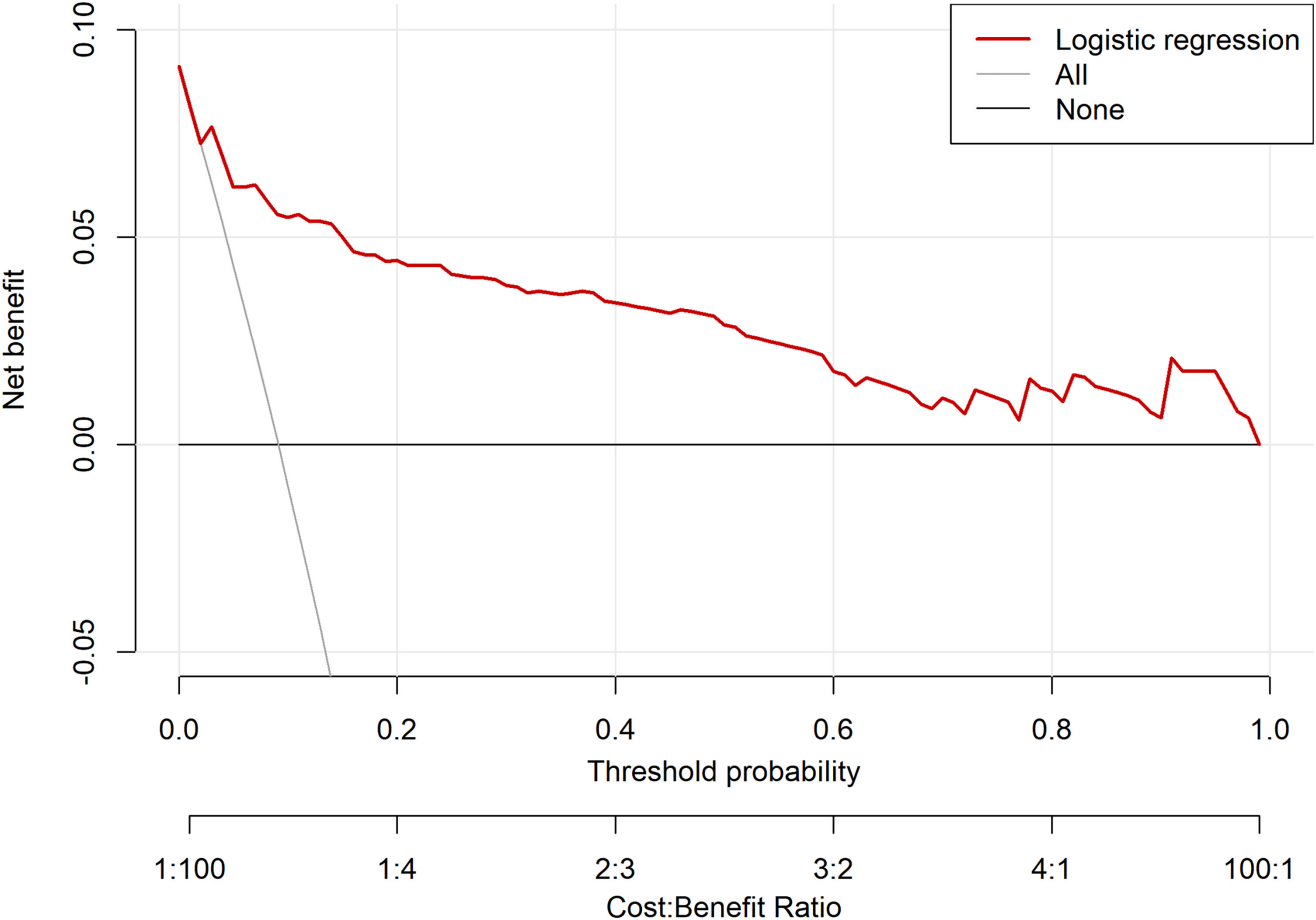
Decision curve analysis for the full-cohort logistic model.

**FIGURE 5:**
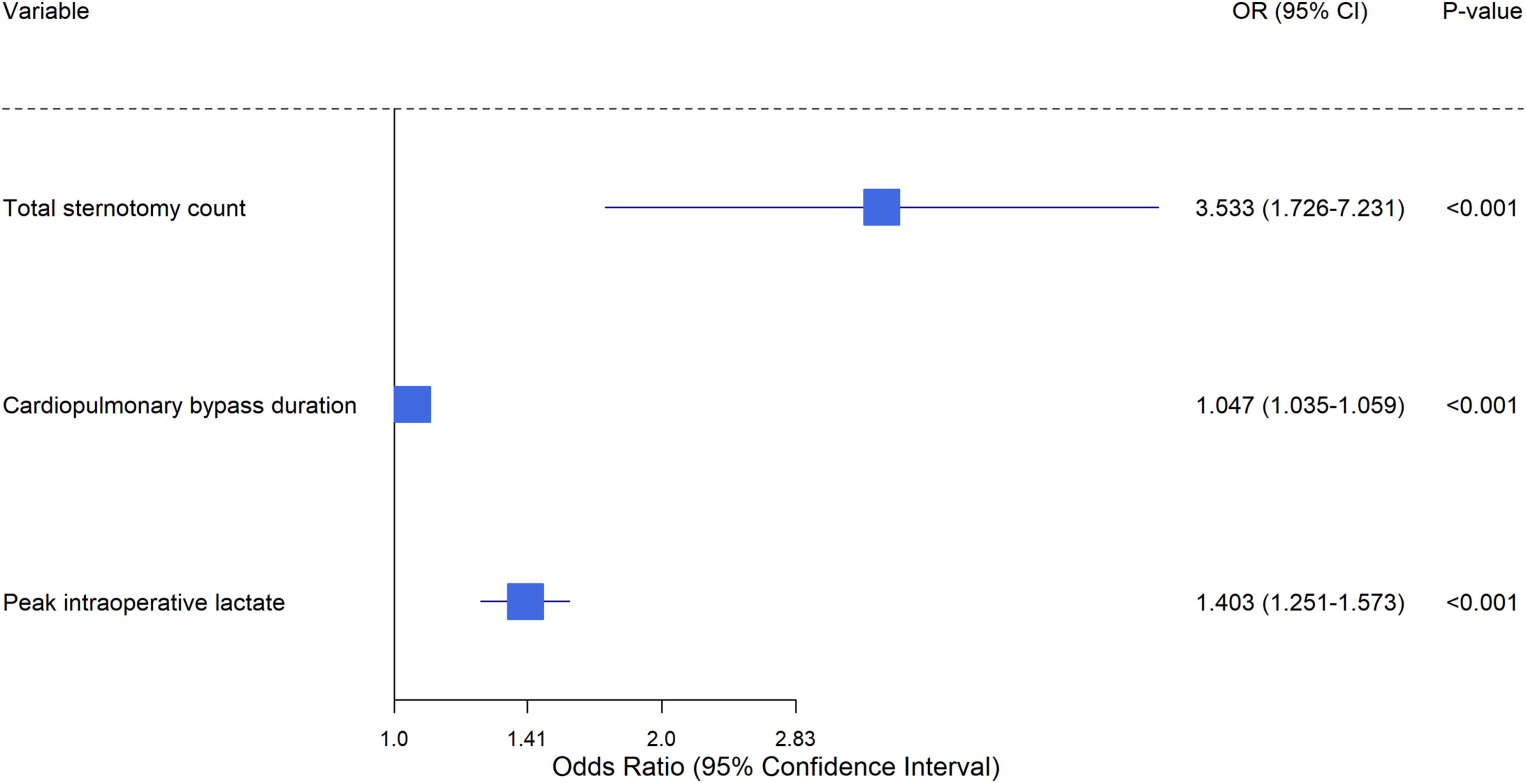
Pooled odds ratios for the final logistic regression model.

The full-cohort apparent AUC was 0.907 (95% CI 0.867–0.946; Figure 2), with an apparent Brier score of 0.050. Bootstrap internal validation showed limited optimism, with an optimism-corrected AUC of 0.903 and optimism-corrected Brier score of 0.052. The optimism-corrected calibration intercept was 0.002, and the optimism-corrected calibratio slope was 0.966. The bootstrap-corrected calibration curve is shown in Figure 3. Decision curve analysis showed positive net benefit compared with treating all or no patients as high risk across a broad range of threshold probabilities in the internal dataset (Figure 4), although these findings require external validation before clinical use. The pooled odds ratios for the final logistic regression model are shown in Figure 5.

The exploratory Youden-index probability cutoff in the full cohort was 0.143. At this internally derived threshold, sensitivity was 77.2% and specificity was 89.1%. This cutoff is reported for research interpretation only and should not be used as a definitive clinical threshold. Detailed bootstrap validation results and the exploratory threshold-based diagnostic performance are provided in Supplementary Tables 6, 7.

### Temporal split sensitivity analysis

**FIGURE 6:**
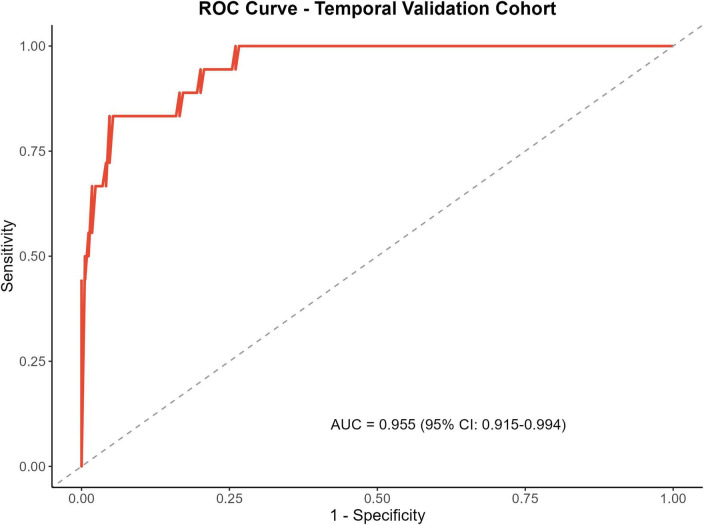
Receiver operating characteristic curve in the temporal validation cohort.

The temporal training cohort included 439 patients admitted from June 9, 2020 to July 28, 2022, with 39 events (8.88%). The temporal validation cohort included 187 patients admitted from August 2, 2022 to June 14, 2023, with 18 events (9.63%). The model AUC was 0.884 (95% CI 0.830–0.938) in the temporal training cohort and 0.955 (95% CI 0.915–0.994) in the temporal validation cohort (Figure 6). The temporal validation Brier score was 0.041, calibration intercept was −0.316, and calibration slope was 1.215. Additional details of the temporal split cohorts, refitted regression model, validation performance, and exploratory threshold analysis are provided in Supplementary Tables 8–11 and Supplementary Figures 1–3. Because total sternotomy count showed limited dispersion in the cohort, its original and binary distributions are additionally summarized in Supplementary Tables 18, 19.

### Complete-case sensitivity analysis

A complete-case sensitivity analysis was performed in the original 600-patient cohort. This cohort excluded 26 non-event patients with incomplete perioperative data under the original complete-case definition, while all 57 events remained in the analysis. Among the excluded patients, 19 had one missing variable, 6 had two missing variables, and 1 had five missing variables.

In this complete-case sensitivity cohort, the refitted three-predictor logistic model produced effect estimates very similar to those of the primary multiple-imputation analysis. Total sternotomy count remained associated with the outcome (OR 3.787, 95% CI 1.827–7.848, *P* < 0.001), as did CPB duration (OR 1.047, 95% CI 1.034–1.059, *P* < 0.001) and peak intraoperative lactate (OR 1.405, 95% CI 1.252–1.577, *P* < 0.001). Apparent discrimination also remained stable, with an AUC of 0.906 (95% CI 0.865–0.942), Brier score of 0.051, calibration intercept approximating 0, and calibration slope of 1.000. Additional details of the original 600-patient complete-case sensitivity analysis are provided in Supplementary Tables 12–15.

### Exploratory machine-learning analyses

**FIGURE 7:**
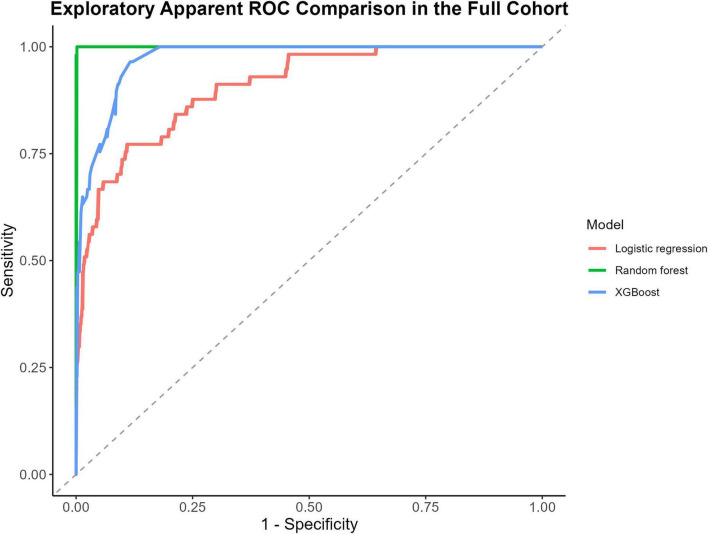
Apparent ROC curves for exploratory logistic regression, random forest, and XGBoost models.

Using the same three predictors in the full 626-patient cohort, repeated cross-validation yielded AUCs of 0.904 for logistic regression, 0.911 for random forest, and 0.933 for XGBoost. Apparent AUCs were 0.907 for logistic regression, 1.000 for random forest, and 0.972 for XGBoost (Figure 7). The near-perfect apparent performance and extreme apparent calibration slope of random forest were consistent with overfitting. Therefore, the machine-learning results are presented as exploratory comparisons only and are not interpreted as evidence of superiority over the logistic model. Detailed comparative performance and variable-importance results for the exploratory machine-learning analyses are provided in Supplementary Tables 16, 17 and Supplementary Figure 4. The exploratory machine-learning results are summarized in Table 3.

**TABLE 3:** Exploratory machine-learning analyses in the 626-patient cohort.

Model	CV AUC	CV SD	Pooled resample AUC (95% CI)	Pooled brier	Apparent AUC	Apparent cal. slope
Logistic regression	0.904	0.039	0.902 (0.884–0.920)	0.051	0.907	1
Random forest	0.911	0.047	0.909 (0.891–0.928)	0.051	1	46.758
XGBoost	0.933	0.033	0.929 (0.914–0.944)	0.046	0.972	1.604

## Discussion

In this retrospective single-center study of patients undergoing CABG with CPB, severe postoperative aminotransferase elevation compatible with HH occurred in approximately 9% of patients and was associated with markedly higher in-hospital mortality. A parsimonious model based on total sternotomy count, CPB duration, and peak intraoperative lactate showed good apparent discrimination and limited optimism after bootstrap internal validation. These predictors are clinically plausible because they reflect operative complexity, duration of extracorporeal circulation, and systemic hypoperfusion burden.

Several analytical choices were used to reduce overfitting and improve internal validity. First, missing data were handled with multiple imputation as the primary analytic approach rather than by excluding patients with partial missingness. Second, the primary model was fitted in the full cohort and internally validated using bootstrap optimism correction, which is more statistically efficient than a single random split in a modest event-count dataset (26). Third, feature selection was based on LASSO penalized logistic regression run separately in each imputed dataset, so that stability could be assessed across imputations rather than inferred from a single completed dataset (25, 26). Fourth, we retained both a temporal split analysis and a complete-case sensitivity analysis, and both yielded findings that were directionally and quantitatively consistent with the primary analysis. Fifth, machine-learning models were treated as exploratory analyses rather than as evidence of clinical superiority.

The selected predictors are consistent with the known pathophysiology of HH, which involves reduced hepatic oxygen delivery, venous congestion, and systemic hypoxemia leading to centrilobular hepatocellular injury and reperfusion-related inflammatory amplification(1, 4, 8, 13–15). CPB duration may capture prolonged exposure to non-physiologic circulation, hemodilution, inflammatory activation, and periods of impaired hepatic perfusion. Peak intraoperative lactate integrates the severity of systemic hypoperfusion and anaerobic metabolism and is increasingly recognized as a perioperative risk marker after cardiac surgery (19–22). Total sternotomy count may mark redo or more complex operative procedures with greater surgical burden and technical difficulty (18). At the same time, total sternotomy count showed limited dispersion in our cohort, with most patients undergoing a single sternotomy; therefore, this predictor should still be interpreted cautiously as a risk marker rather than a causal factor, even though its direction of association was clinically plausible and its LASSO selection stability remained moderate to high.

Compared with prior literature, the main contribution of the present study is not the use of a more complex algorithm, but the focus on a specific and underexplored perioperative setting. Published studies on HH and related liver injury have largely involved mixed ICU populations, extracorporeal cardiopulmonary resuscitation, heart failure cohorts, or selected aortic procedures rather than routine CABG performed with CPB (5, 6, 9, 13, 30–32). Similarly, existing prediction work has emphasized mortality among patients with established HH or ischemic liver injury after other cardiac or aortic operations rather than early recognition of HH-compatible postoperative liver injury in CABG patients (9, 32). In that context, a simple three-predictor model for CABG with CPB may be clinically appealing if it can be externally validated and recalibrated in broader settings.

Diagnostic thresholds for HH vary across studies, and the syndrome is usually defined by marked aminotransferase elevation, an appropriate hypoxic or hemodynamic context, and exclusion of alternative causes (1–4, 10–14, 16). We therefore used a 20-times-upper-limit operational threshold to capture severe hepatocellular injury while excluding patients with clear competing hepatic diagnoses during data extraction. Nevertheless, because structured hemodynamic variables such as shock, low cardiac output, or mechanical circulatory support were not uniformly available and no blinded adjudication committee was convened, the endpoint should be interpreted as severe postoperative aminotransferase elevation compatible with HH rather than as a prospectively adjudicated HH diagnosis. When diagnostic uncertainty materially changes management, liver biopsy can document centrilobular necrosis, but biopsy is usually avoided in critically ill or coagulopathic patients (10, 11, 33–34). This distinction is important when interpreting both the apparent discrimination and the possibility of residual misclassification.

The model is not a preoperative risk-stratification tool because CPB duration and peak lactate are available intraoperatively or immediately after separation from bypass. Its most plausible application is early perioperative risk awareness: identifying patients who may warrant closer postoperative liver biochemistry monitoring, careful reassessment of perfusion and oxygenation, and intensified supportive care. No specific pharmacotherapy exists for HH, and management remains focused on correcting the precipitating circulatory or respiratory disturbance and supporting failing organs (15). Accordingly, the present model should be understood as a pragmatic aid to surveillance and early recognition rather than as a trigger for a validated treatment pathway. The present study did not test whether model-guided management improves outcomes, so clinical utility remains hypothetical.

The exploratory machine-learning results also require cautious interpretation. Although XGBoost achieved the highest cross-validated AUC, the analyses used only three predictors, which limits the opportunity for machine-learning methods to exploit complex non-linear or high-dimensional structure. In addition, the random forest model showed near-perfect apparent performance, including an apparent AUC of 1.000 and an extreme calibration slope, which is a classic signal of overfitting in small event-count prediction studies. For this reason, the logistic model remains the preferred primary model because it is transparent, parsimonious, more readily interpretable at the bedside, and better matched to the available sample size.

The internal validation strategy also deserves emphasis. Bootstrap optimism correction served as the primary internal validation approach because it preserves the full cohort for model development and provides a more efficient estimate of likely optimism than a single random split (26). We retained a temporal split as a secondary sensitivity analysis, and the model continued to show good discrimination in the later cohort. However, this temporal split still represents internal validation within one center and should not be interpreted as proof of transportability.

Taken together, these findings suggest that a simple perioperative model can capture a clinically meaningful signal of HH-compatible postoperative liver injury after CPB-assisted CABG. In this sense, the study contributes a focused and clinically interpretable framework for early risk awareness in a setting where dedicated prediction tools remain limited. Future work should prioritize multicenter validation and evaluate whether richer perioperative trajectories, such as lactate clearance or serial aminotransferase dynamics, further improve discrimination, calibration, and clinical usefulness.

### Limitations

Several limitations deserve emphasis. First, the study was retrospective and single-center, so differences in patient mix, surgical practice, CPB management, laboratory monitoring, and postoperative care may limit transportability. External validation is essential before broader implementation. Second, the outcome definition was operational and based primarily on severe postoperative aminotransferase elevation after exclusion of alternative causes during data extraction. A prospective blinded adjudication committee and inter-rater reliability assessment were not available, so misclassification bias remains possible. Third, incorporation bias cannot be fully excluded because CPB duration and lactate are conceptually related to perioperative hypoperfusion, even though they were not used as formal components of the biochemical outcome threshold. This overlap may inflate associations and internal performance. Fourth, there were only 57 events, and no formal prospective sample-size calculation was performed before data collection. The event count constrained model complexity and makes machine-learning analyses particularly vulnerable to overfitting. Fifth, despite low missingness and use of multiple imputation, the missing-data mechanism cannot be proven in a retrospective dataset. Sixth, the Youden-index threshold was internally derived and should be considered exploratory rather than a clinical decision cutoff. Finally, only in-hospital outcomes were available, and the long-term prognostic impact of the HH-compatible outcome could not be assessed.

## Conclusion

We developed and internally validated a three-predictor model for severe postoperative aminotransferase elevation compatible with HH after CABG with CPB. Total sternotomy count, CPB duration, and peak intraoperative lactate were consistently associated with the outcome. The model may support early perioperative risk awareness within the study setting, but it is not a preoperative risk tool and should not be implemented clinically without external validation.

## Data Availability

The raw data supporting the conclusions of this article will be made available by the authors, without undue reservation.
