# Supplementary material for: Development and internal validation of a prediction model for hypoxic hepatitis after coronary artery bypass grafting with cardiopulmonary bypass
Source: Front Med (Lausanne). 2026 Jun 8;13:1785046. doi: 10.3389/fmed.2026.1785046 (PMC13283789; doi:10.3389/fmed.2026.1785046)

**Supplementary Materials**

# Supplementary Tables

**Supplementary Table 1. Pattern of missing predictor data in the full cohort.**

| Predictor | Missing, n | Missing, % |
| --- | --- | --- |
| NYHA class >= III | 8 | 1.28% |
| Height | 7 | 1.12% |
| Preoperative INR | 6 | 0.96% |
| Preoperative ALP | 3 | 0.48% |
| Preoperative GGT | 3 | 0.48% |
| Preoperative LVEF | 3 | 0.48% |
| Weight | 2 | 0.32% |
| Preoperative ALT | 1 | 0.16% |
| Preoperative AST | 1 | 0.16% |
| Preoperative TBIL | 1 | 0.16% |

*Note.* No missing values were present for the outcome, CPB duration, peak intraoperative lactate, or total sternotomy count.

**Supplementary Table 2. Descriptive characteristics by outcome status in the first completed imputed dataset.**

| Variable | Overall | No HH-compatible outcome | HH-compatible outcome | P value |
| --- | --- | --- | --- | --- |
| Patients, n | 626 | 569 | 57 |  |
| Age, years, median [IQR] | 63.00 [56.00, 68.00] | 62.00 [56.00, 68.00] | 65.00 [58.00, 70.00] | 0.221 |
| Height, m, median [IQR] | 1.68 [1.61, 1.72] | 1.68 [1.62, 1.72] | 1.65 [1.60, 1.72] | 0.254 |
| Weight, kg, median [IQR] | 71.00 [63.00, 78.00] | 71.00 [63.00, 79.00] | 68.00 [65.00, 77.00] | 0.267 |
| BMI, kg/m2, median [IQR] | 25.40 [23.39, 27.50] | 25.38 [23.42, 27.55] | 25.53 [22.86, 27.06] | 0.823 |
| Female sex, n (%) | 140 (22.4) | 125 (22.0) | 15 (26.3) | 0.559 |
| Smoking history, n (%) | 305 (48.7) | 273 (48.0) | 32 (56.1) | 0.300 |
| Alcohol use, n (%) | 172 (27.5) | 157 (27.6) | 15 (26.3) | 0.960 |
| Total sternotomy count, median [IQR] | 1.00 [1.00, 1.00] | 1.00 [1.00, 1.00] | 1.00 [1.00, 2.00] | <0.001 |
| Hypertension, n (%) | 416 (66.5) | 376 (66.1) | 40 (70.2) | 0.633 |
| Diabetes mellitus, n (%) | 266 (42.5) | 239 (42.0) | 27 (47.4) | 0.522 |
| Hyperlipidemia, n (%) | 353 (56.4) | 314 (55.2) | 39 (68.4) | 0.075 |
| Lung disease, n (%) | 79 (12.6) | 72 (12.7) | 7 (12.3) | 1.000 |
| NYHA class >= III, n (%) | 306 (48.9) | 272 (47.8) | 34 (59.6) | 0.117 |
| Old myocardial infarction, n (%) | 317 (50.6) | 287 (50.4) | 30 (52.6) | 0.860 |
| Left main disease, n (%) | 131 (20.9) | 118 (20.7) | 13 (22.8) | 0.845 |
| Peripheral arterial disease, n (%) | 310 (49.5) | 270 (47.5) | 40 (70.2) | 0.002 |
| Number of grafts, median [IQR] | 3.00 [3.00, 4.00] | 3.00 [3.00, 4.00] | 3.00 [3.00, 4.00] | 0.719 |
| Intraoperative adverse events, n (%) | 130 (20.8) | 109 (19.2) | 21 (36.8) | 0.003 |
| Peak intraoperative lactate, mmol/L, median [IQR] | 3.75 [2.40, 6.71] | 3.30 [2.25, 6.15] | 7.07 [5.80, 9.93] | <0.001 |
| CPB duration, min, median [IQR] | 116.50 [95.00, 142.75] | 113.00 [91.00, 138.00] | 171.00 [137.00, 182.00] | <0.001 |
| Preoperative LVEF, %, median [IQR] | 58.00 [50.00, 63.00] | 58.00 [50.00, 63.00] | 55.00 [42.00, 61.00] | 0.151 |
| Preoperative ALT, U/L, median [IQR] | 21.00 [14.00, 31.00] | 21.00 [14.00, 31.00] | 20.00 [14.00, 29.00] | 0.613 |
| Preoperative AST, U/L, median [IQR] | 18.00 [15.00, 24.75] | 18.00 [15.00, 24.00] | 19.00 [15.00, 27.00] | 0.478 |
| Preoperative albumin, g/L, mean (SD) | 42.69 (3.67) | 42.72 (3.65) | 42.34 (3.92) | 0.460 |
| Preoperative ALP, U/L, median [IQR] | 75.00 [63.00, 89.00] | 75.00 [63.00, 88.00] | 73.00 [63.00, 93.00] | 0.958 |
| Preoperative GGT, U/L, median [IQR] | 26.50 [19.00, 40.00] | 26.00 [19.00, 40.00] | 28.00 [18.00, 44.00] | 0.794 |
| Preoperative TBIL, umol/L, median [IQR] | 10.44 [7.70, 14.00] | 10.51 [7.73, 14.10] | 9.74 [7.05, 13.63] | 0.553 |
| Preoperative INR, median [IQR] | 1.01 [0.97, 1.05] | 1.01 [0.97, 1.05] | 1.01 [0.97, 1.05] | 0.841 |
| Hospital length of stay, days, median [IQR] | 15.00 [11.00, 19.00] | 15.00 [11.00, 19.00] | 18.00 [12.00, 24.00] | 0.005 |
| ICU length of stay, days, median [IQR] | 3.00 [2.00, 5.00] | 3.00 [2.00, 4.00] | 8.00 [4.00, 11.00] | <0.001 |
| In-hospital mortality, n (%) | 54 (8.6) | 26 (4.6) | 28 (49.1) | <0.001 |

*Note.* This table is descriptive and is shown using the first completed imputed dataset for presentation only. Primary regression estimates were pooled across 20 imputed datasets using Rubin's rules.

**Supplementary Table 3. LASSO selection stability across 20 imputed datasets.**

| Variable | lambda.1se | lambda.min | Stability interpretation | Retained |
| --- | --- | --- | --- | --- |
| CPB duration, per minute | 20/20 (100%) | 20/20 (100%) | Selected in all imputed datasets under lambda.1se | Yes |
| Peak intraoperative lactate, per mmol/L | 20/20 (100%) | 20/20 (100%) | Selected in all imputed datasets under lambda.1se | Yes |
| Total sternotomy count | 15/20 (75%) | 20/20 (100%) | Selected in most imputed datasets under lambda.1se | Yes |
| Peripheral arterial disease | 0/20 (0%) | 6/20 (30%) | Rarely selected; not retained | No |
| Hyperlipidemia | 0/20 (0%) | 3/20 (15%) | Rarely selected; not retained | No |

*Note.* LASSO penalized logistic regression was run separately in each imputed dataset with 10-fold cross-validation. Selection stability was assessed across imputations rather than from a single completed dataset.

**Supplementary Table 4. Pooled multivariable logistic regression results after multiple imputation.**

| Predictor | OR | Lower 95% CI | Upper 95% CI | P value |
| --- | --- | --- | --- | --- |
| Total sternotomy count | 3.533 | 1.724 | 7.241 | <0.001 |
| CPB duration, per minute | 1.047 | 1.035 | 1.059 | <0.001 |
| Peak intraoperative lactate, per mmol/L | 1.403 | 1.251 | 1.574 | <0.001 |

**Supplementary Table 5. Variance inflation factors for the final predictors.**

| Predictor | VIF |
| --- | --- |
| Total sternotomy count | 1.055 |
| CPB duration, per minute | 1.020 |
| Peak intraoperative lactate, per mmol/L | 1.070 |

*Note.* VIF values were estimated in the first completed imputed dataset because multicollinearity diagnostics are descriptive rather than pooled inferential parameters.

**Supplementary Table 6. Bootstrap internal validation summary for the primary model.**

| Metric | Apparent | Mean optimism | Optimism-corrected |
| --- | --- | --- | --- |
| AUC | 0.907 | 0.003 | 0.903 |
| Brier score | 0.050 | -0.002 | 0.052 |
| Calibration intercept | -0.000 | -0.002 | 0.002 |
| Calibration slope | 1.000 | 0.034 | 0.966 |

*Note.* Internal validation used 1000 bootstrap resamples.

**Supplementary Table 7. Diagnostic performance at the exploratory probability threshold in the full cohort.**

| Threshold method | Threshold | Sensitivity | Specificity | PPV | NPV | Accuracy |
| --- | --- | --- | --- | --- | --- | --- |
| Youden index from the full cohort ROC (exploratory only) | 0.143 | 0.772 | 0.891 | 0.415 | 0.975 | 0.880 |

*Note.* The cutoff was derived using the Youden index and is reported for exploratory interpretation only, not as a recommended clinical decision threshold.

**Supplementary Table 8. Temporal split cohort summary.**

| Cohort | Start date | End date | N | Events | Event rate |
| --- | --- | --- | --- | --- | --- |
| Temporal training cohort | 2020-06-09 | 2022-07-28 | 439 | 39 | 8.88% |
| Temporal validation cohort | 2022-08-02 | 2023-06-14 | 187 | 18 | 9.63% |

**Supplementary Table 9. Refit multivariable logistic regression model in the temporal training cohort.**

| Predictor | OR | Lower 95% CI | Upper 95% CI | P value |
| --- | --- | --- | --- | --- |
| Total sternotomy count | 3.432 | 1.226 | 9.606 | 0.019 |
| CPB duration, per minute | 1.045 | 1.031 | 1.059 | <0.001 |
| Peak intraoperative lactate, per mmol/L | 1.371 | 1.203 | 1.564 | <0.001 |

**Supplementary Table 10. Model performance in the temporal split sensitivity analysis.**

| Cohort | AUC | 95% CI | Brier score | Calibration intercept | Calibration slope |
| --- | --- | --- | --- | --- | --- |
| Training | 0.884 | 0.830-0.938 | 0.054 | -0.000 | 1.000 |
| Validation | 0.955 | 0.915-0.994 | 0.041 | -0.316 | 1.215 |

**Supplementary Table 11. Diagnostic performance at the exploratory temporal-validation threshold.**

| Threshold method | Threshold | Sensitivity | Specificity | PPV | NPV | Accuracy |
| --- | --- | --- | --- | --- | --- | --- |
| Youden index from temporal training ROC (exploratory only) | 0.152 | 0.833 | 0.893 | 0.455 | 0.981 | 0.888 |

*Note.* The threshold was derived from the temporal training ROC curve and then examined in the temporal validation cohort. It remains exploratory only.

**Supplementary Table 12. Cohort summary for the original 600-patient complete-case sensitivity analysis.**

| Analysis | Full cohort n | Complete-case n | Excluded for missing | Excluded non-events | Excluded events | Events retained | Event rate |
| --- | --- | --- | --- | --- | --- | --- | --- |
| Original 600-patient complete-case cohort | 626 | 600 | 26 | 26 | 0 | 57 | 9.50% |

*Note.* This sensitivity analysis used the original 600-patient complete-case cohort retained from the initial data-cleaning workflow for consistency with the earlier manuscript version. All 57 events remained in the complete-case cohort.

**Supplementary Table 13. Pattern of missing variables among patients excluded from the original 600-patient complete-case cohort.**

| Missing variables, n | Patients, n |
| --- | --- |
| 1 | 19 |
| 2 | 6 |
| 5 | 1 |

**Supplementary Table 14. Multivariable logistic regression results in the original 600-patient complete-case sensitivity analysis.**

| Predictor | OR | Lower 95% CI | Upper 95% CI | P value |
| --- | --- | --- | --- | --- |
| Total sternotomy count | 3.787 | 1.827 | 7.848 | <0.001 |
| CPB duration, per minute | 1.047 | 1.034 | 1.059 | <0.001 |
| Peak intraoperative lactate, per mmol/L | 1.405 | 1.252 | 1.577 | <0.001 |

**Supplementary Table 15. Apparent model performance in the original 600-patient complete-case sensitivity analysis.**

| Analysis | AUC | 95% CI | Brier score | Calibration intercept | Calibration slope |
| --- | --- | --- | --- | --- | --- |
| Original 600-patient complete-case cohort | 0.906 | 0.865-0.942 | 0.051 | -0.000 | 1.000 |

**Supplementary Table 16. Comparative performance of logistic regression, random forest, and XGBoost in the full 626-patient cohort.**

| Model | Mean CV AUC | Pooled resample AUC (95% CI) | Pooled resample Brier | Apparent AUC (95% CI) | Apparent Brier | Apparent intercept | Apparent slope |
| --- | --- | --- | --- | --- | --- | --- | --- |
| Logistic regression | 0.904 | 0.902 (0.884-0.920) | 0.051 | 0.907 (0.867-0.946) | 0.050 | -0.000 | 1.000 |
| Random forest | 0.911 | 0.909 (0.891-0.928) | 0.051 | 1.000 (1.000-1.000) | 0.008 | -3.68e+15 | 46.758 |
| XGBoost | 0.933 | 0.929 (0.914-0.944) | 0.046 | 0.972 (0.959-0.985) | 0.038 | -0.061 | 1.604 |

*Note.* Mean CV AUC corresponds to the repeated cross-validation discrimination reported in the main Results. Pooled resample AUC with 95% CI is shown separately. Machine-learning analyses were exploratory because only three predictors were used and the event count was modest; apparent performance is shown to illustrate overfitting risk and should not be interpreted as model superiority.

**Supplementary Table 17. Variable importance in the exploratory machine-learning models.**

| Variable | Random forest importance | XGBoost importance |
| --- | --- | --- |
| CPB duration, per minute | 41.794 | 0.481 |
| Peak intraoperative lactate, per mmol/L | 28.187 | 0.452 |
| Total sternotomy count | 5.984 | 0.067 |

**Supplementary Table 18. Distribution of total sternotomy count and corresponding HH-compatible outcomes.**

| Total sternotomy count | Overall, n (%) | HH-compatible outcome, n (%) | No HH-compatible outcome, n (%) |
| --- | --- | --- | --- |
| 1 | 564 (90.1%) | 38 (66.7%) | 526 (92.4%) |
| 2 | 53 (8.5%) | 14 (24.6%) | 39 (6.9%) |
| 3 | 9 (1.4%) | 5 (8.8%) | 4 (0.7%) |

**Supplementary Table 19. Binary distribution of total sternotomy count.**

| Sternotomy category | Overall, n (%) | HH-compatible outcome, n (%) | No HH-compatible outcome, n (%) |
| --- | --- | --- | --- |
| 1 | 564 (90.1%) | 38 (66.7%) | 526 (92.4%) |
| >=2 | 62 (9.9%) | 19 (33.3%) | 43 (7.6%) |

# Supplementary Figures

**Supplementary Figure 1. ROC curve for the temporal training cohort.**


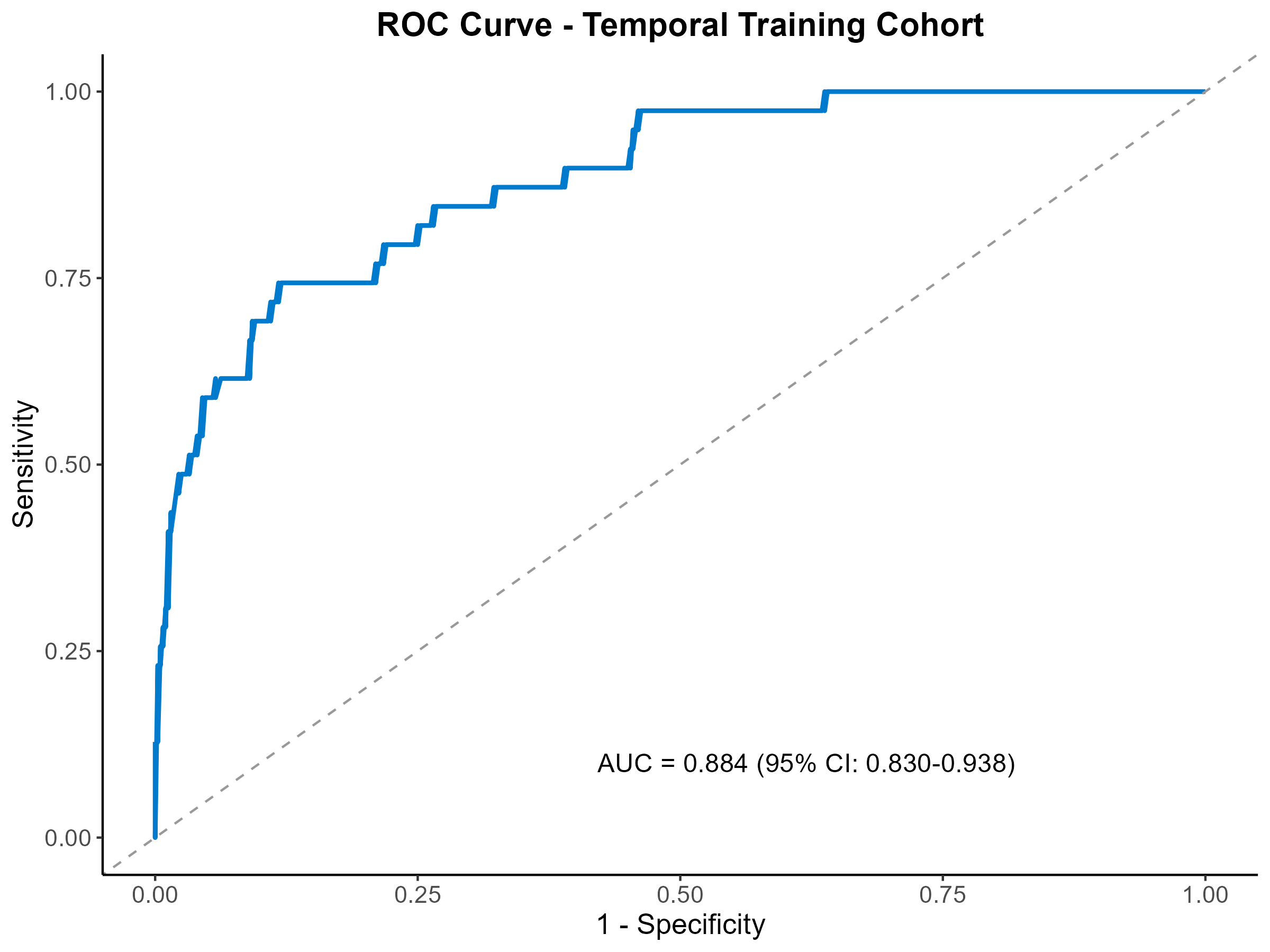


**Supplementary Figure 2. Calibration plot for the temporal validation cohort.**


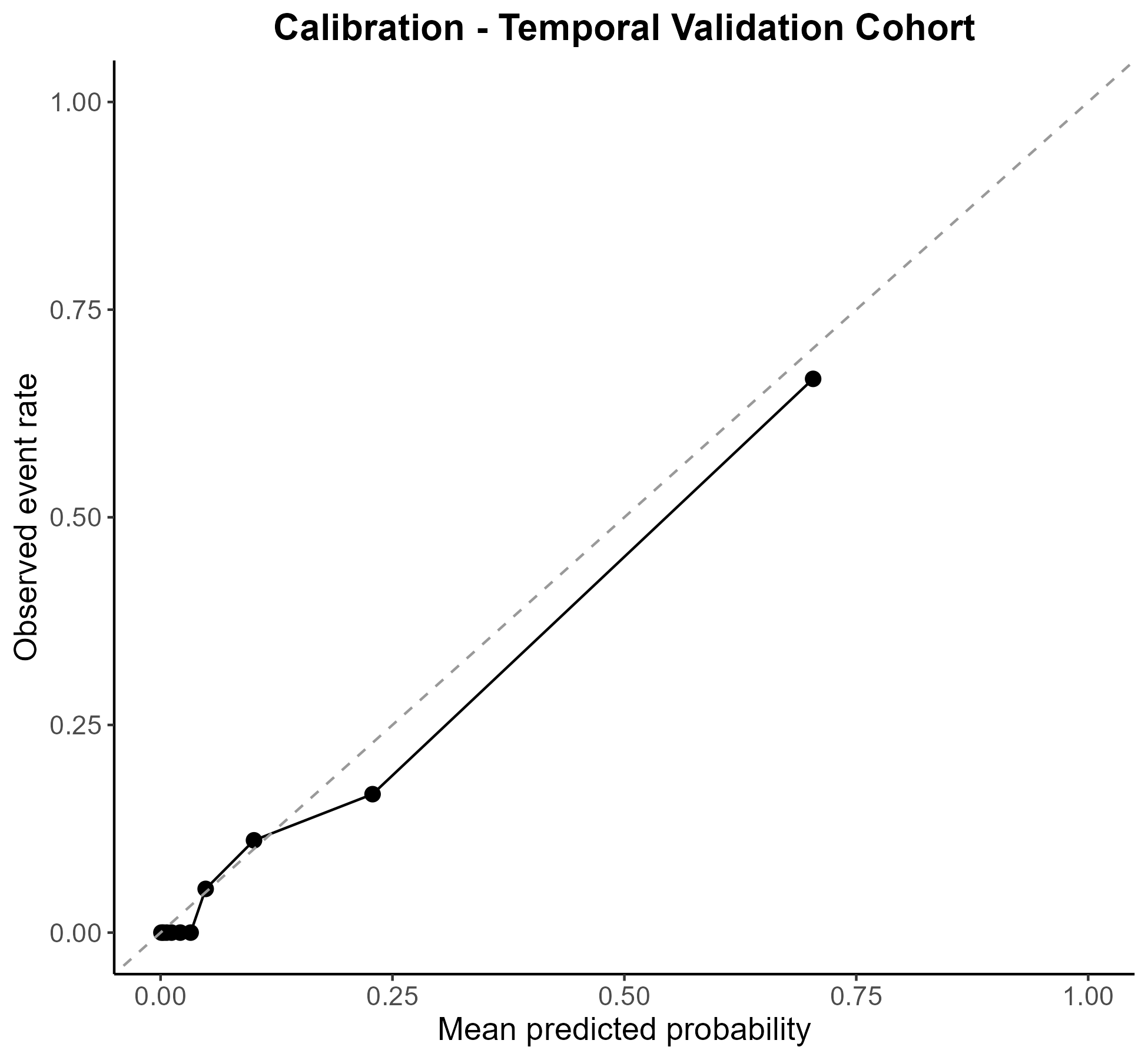


**Supplementary Figure 3. Decision curve analysis for the temporal validation cohort.**


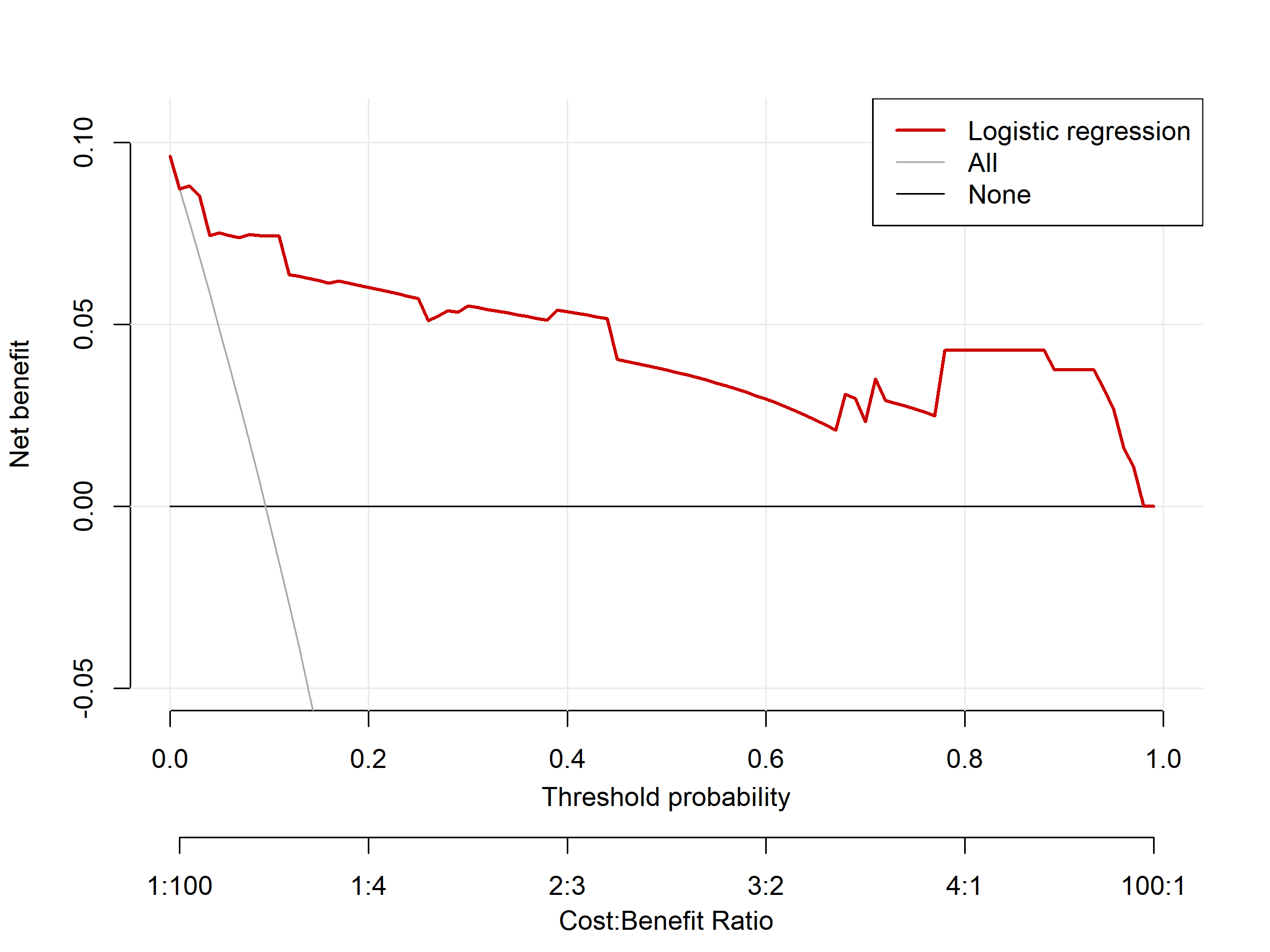


**Supplementary Figure 4. Resampled AUC distributions from repeated cross-validation for the exploratory machine-learning analyses.**


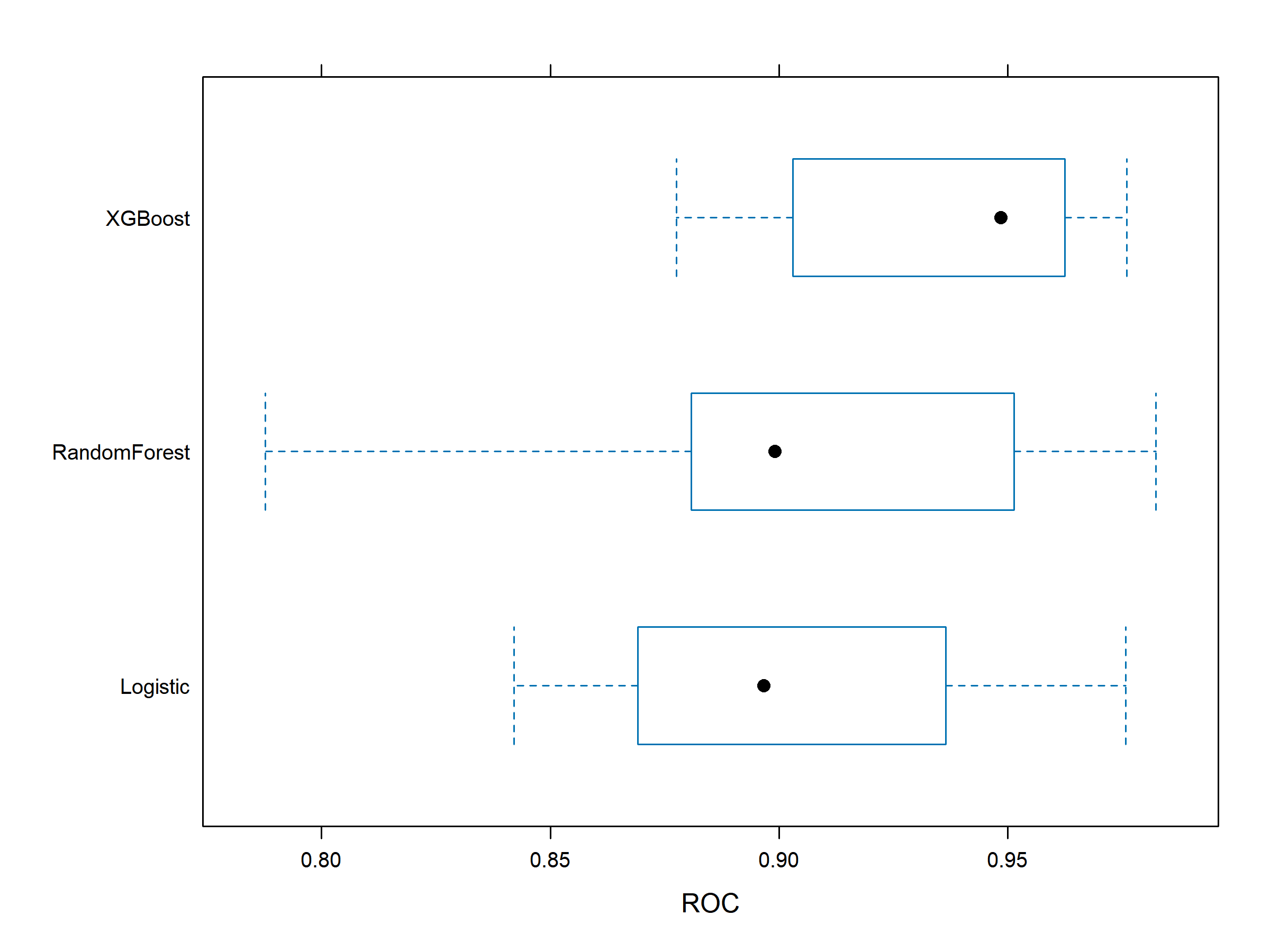

Supplement: Supplementary file 1 [file Data_Sheet_1.docx]
