# Supplementary material for: Development and internal validation of a prediction model for hypoxic hepatitis after coronary artery bypass grafting with cardiopulmonary bypass
Source: Front Med (Lausanne). 2026 Jun 8;13:1785046. doi: 10.3389/fmed.2026.1785046 (PMC13283789; doi:10.3389/fmed.2026.1785046)
Supplement: Supplementary file 2 [file Data_Sheet_2.docx]

**PROBAST+AI Assessment**

**Prediction model for postoperative hypoxic hepatitis after cardiopulmonary bypass-assisted CABG**

*Prepared as a separate supplementary file for peer review*

| **Study type** | Combination study under PROBAST+AI: model development with internal evaluation |
| --- | --- |
| **Index model** | Logistic regression-based nomogram for postoperative hypoxic hepatitis |
| **Comparator models** | XGBoost and random forest for comparative internal performance assessment |
| **Overall judgement** | High concern for model development quality; high risk of bias for model evaluation; low applicability concern |

**Step 1. PICOTS**

| **Element** | **Assessment** |
| --- | --- |
| **Population** | Adult patients undergoing cardiopulmonary bypass-assisted coronary artery bypass grafting at a single tertiary cardiac center. |
| **Index model(s)** | A logistic regression-based prediction model and nomogram for postoperative hypoxic hepatitis after on-pump CABG, including total sternotomy count, peak intraoperative lactate level, and cardiopulmonary bypass duration. |
| **Comparator model(s)** | XGBoost and random forest models developed using the same final predictor set for comparative internal performance assessment. |
| **Outcome(s)** | Postoperative hypoxic hepatitis during the index hospitalization. |
| **Timing** | Prediction intended intraoperatively or immediately after separation from cardiopulmonary bypass; prediction horizon limited to the postoperative in-hospital period. |
| **Setting and intended use** | Single-center retrospective tertiary cardiac surgery setting; intended for exploratory perioperative risk stratification and early postoperative surveillance rather than stand-alone clinical decision-making. |

**Step 2. Classification of prediction model assessment**

This study was classified as a combination study because it involved model development and evaluation within the same investigation, including random 7:3 internal validation, temporal split sensitivity analysis, and comparative internal performance assessment against XGBoost and random forest.

**Step 3. Model development assessment**

**Domain 1. Participants and data sources**

| **Judgement** | Low concern |
| --- | --- |
| **Rationale** | The study used a clearly defined retrospective single-center cohort of adults undergoing on-pump CABG in a relevant tertiary cardiac surgical setting. However, the single-center design and exclusion of patients with incomplete clinical data may limit representativeness. |

**Data source and selection**

Retrospective cohort study conducted at Beijing Anzhen Hospital, Capital Medical University. A total of 626 patients were screened, and 600 were included in the primary complete-case analysis after exclusion of 26 patients with incomplete clinical data.

**Signalling questions**

Appropriate data sources: Probably yes. Appropriate study design: Probably yes. Representative dataset after inclusions and exclusions: Probably yes.

**Applicability**

Low concern. The source population and clinical setting align well with the intended use of perioperative risk stratification after cardiopulmonary bypass-assisted CABG.

**Domain 2. Predictors**

| **Judgement** | Low concern |
| --- | --- |
| **Rationale** | Predictors were routinely collected perioperative variables, clearly defined, consistently processed, and available at the intended time of use. |

**Predictors in the final model**

Total sternotomy count, peak intraoperative lactate level, and cardiopulmonary bypass duration.

**Definition and timing**

Total sternotomy count was defined as the total number of sternotomy procedures including the index CABG operation. Peak intraoperative lactate and CPB duration were obtained during the index surgery. All predictors were available intraoperatively or immediately after separation from bypass.

**Signalling questions**

Predictors defined and assessed similarly for all participants: Yes. Pre-processing similar for all participants: Yes. Predictor assessment without formal knowledge of outcome: Probably yes. Predictors available at intended time of use: Yes.

**Applicability**

Low concern. The predictors directly match the intended perioperative clinical context and can be obtained in routine practice.

**Domain 3. Outcome**

| **Judgement** | Low concern |
| --- | --- |
| **Rationale** | The outcome was based on predefined biochemical and clinical criteria and assessed consistently during the same hospitalization. Some residual misclassification remains possible because hepatic perfusion was not directly measured and histopathological confirmation was not routinely available. |

**Outcome definition**

Postoperative hypoxic hepatitis during the index hospitalization, defined using established biochemical and clinical criteria, including marked aminotransferase elevation, an appropriate hypoxic or hemodynamic context, and exclusion of alternative causes.

**Time interval**

Predictors were assessed intraoperatively, and the outcome was determined during the postoperative in-hospital period after CABG.

**Signalling questions**

Outcomes defined and assessed appropriately: Probably yes. Outcomes assessed similarly for all participants: Yes. Outcome assessment without strict blinding to predictors: Probably yes. Time interval appropriate: Yes.

**Applicability**

Low concern. The outcome is aligned with the intended purpose of the prediction model.

**Domain 4. Analysis**

| **Judgement** | High concern |
| --- | --- |
| **Rationale** | The analysis domain was judged as high concern because the number of outcome events was limited relative to the multistep predictor-selection process, the primary analysis relied on complete-case exclusion, internal validation was based mainly on data splitting rather than full optimism-corrected bootstrap resampling, and the model remained internally validated only. Sparse-data concerns were also present for total sternotomy count. |

**Sample size and events**

A total of 600 patients were included in the primary analysis, of whom 57 developed postoperative HH. The training cohort contained 39 HH events, giving an events-per-variable ratio of 13 for the final three-predictor model.

**Model development process**

Candidate predictors were prespecified before model fitting on the basis of routine perioperative availability, clinical plausibility, and prior literature. LASSO regression and the Boruta algorithm were applied to the same prespecified candidate set, and only predictors retained by both methods were carried forward into the final multivariable logistic regression model and nomogram.

**Performance measures**

Model performance was assessed using ROC AUC, calibration, and decision curve analysis. Internal validation was performed using a random 7:3 split, and temporal split sensitivity analysis was conducted. An exploratory threshold was derived using the Youden index.

**Missing data**

Missingness was low overall and no missing outcome data were observed. The primary analysis used a complete-case approach, excluding 26 patients with incomplete clinical data. Random forest multiple imputation was performed as a sensitivity analysis, and pooled estimates were obtained using Rubin's rules.

**Signalling questions**

Reasonable sample size: Probably no. Appropriate handling of continuous and categorical predictors: Yes. Appropriate handling of missing data: Probably no. Measures to address overfitting: Probably yes. Class imbalance adjustment: Not applicable.

**Applicability**

Low concern. The analytical concerns primarily affect risk of bias rather than the clinical relevance of the question addressed.

**Step 3. Model evaluation assessment**

Because this was a combination study, model evaluation was assessed separately for risk of bias using the same four PROBAST+AI domains.

**Domain 1. Participants and data sources**

| **Judgement** | Low risk of bias |
| --- | --- |
| **Rationale** | The evaluation cohorts came from the same single-center retrospective source population. This is acceptable for internal evaluation but limits transportability beyond similar settings. |

**Evaluation cohorts**

Internal evaluation was based on a 7:3 random split into a training cohort of 420 patients and a testing cohort of 180 patients. A temporal split sensitivity analysis was also performed using admission date.

**Signalling questions**

Appropriate data source: Probably yes. Appropriate study design: Probably yes. Representative evaluation dataset after exclusions: Probably yes.

**Applicability**

Low concern.

**Domain 2. Predictors**

| **Judgement** | Low risk of bias |
| --- | --- |
| **Rationale** | Predictors were consistently defined, assessed in the same way across cohorts, and available at the time the model was intended to be used. |

**Predictor consistency**

The same three predictors were used for model evaluation: total sternotomy count, peak intraoperative lactate, and CPB duration.

**Signalling questions**

Predictors defined consistently: Yes. Pre-processing consistent: Yes. Assessment without formal blinding: Probably yes. Available at time of intended use: Yes.

**Applicability**

Low concern.

**Domain 3. Outcome**

| **Judgement** | Low risk of bias |
| --- | --- |
| **Rationale** | Outcome assessment followed the same predefined HH criteria across the full cohort and validation subsets. |

**Outcome consistency**

HH was adjudicated according to the same biochemical and clinical criteria in both the random-split cohorts and the temporal split sensitivity analysis.

**Signalling questions**

Appropriate definition and assessment: Probably yes. Similar assessment for all participants: Yes. Assessment without strict blinding to predictors: Probably yes. Appropriate time interval: Yes.

**Applicability**

Low concern.

**Domain 4. Analysis**

| **Judgement** | High risk of bias |
| --- | --- |
| **Rationale** | The evaluation analysis domain was judged as high risk of bias because internal validation relied mainly on data splitting with limited numbers of outcome events, complete-case exclusion was used in the primary analysis, no full optimism-corrected bootstrap validation was reported, and all evaluations remained within a single-center retrospective dataset. |

**Validation events**

The testing cohort contained 18 HH events. In the temporal validation cohort, 18 HH events were also observed.

**Evaluation metrics**

Performance measures included AUC with 95% confidence intervals, calibration, Brier score, decision curve analysis, and pairwise DeLong tests in comparative model analyses.

**Missing data handling**

Participants with incomplete clinical data were excluded from the primary analysis. Random forest multiple imputation was used only as a sensitivity analysis.

**Signalling questions**

Avoidance of apparent performance only: Yes for internal evaluation. Reasonable sample size for evaluation: Probably no. Appropriate handling of missing data: Probably no. Evidence against data leakage in data splitting: Probably yes. Appropriate evaluation of calibration, discrimination, and net benefit: Probably yes.

**Applicability**

Low concern.

**Step 4. Overall judgement**

| **Section** | **Overall judgement** | **Summary** |
| --- | --- | --- |
| **Model development quality** | **High concern** | Driven mainly by limitations in the analysis domain, including limited event numbers, complete-case primary analysis, multistep predictor selection, sparse-data concerns for total sternotomy count, and the absence of bootstrap-based optimism correction. |
| **Model evaluation risk of bias** | **High** | Driven mainly by the retrospective single-center design, limited validation events, primary use of complete-case analysis, and reliance on internal rather than external validation. |
| **Applicability concern** | **Low** | The study population, predictors, outcome, and intended clinical context were well aligned with exploratory perioperative HH risk stratification after on-pump CABG. |

**Notes for submission**

This assessment was prepared as a separate supplementary document to accompany the revised manuscript. The judgement of high concern and high risk of bias was retained intentionally to reflect the limited number of outcome events, the primary complete-case analysis, sparse-data concerns for total sternotomy count, and the fact that all validation procedures remained internal to a single-center retrospective dataset.

These ratings do not indicate that the study lacks value. Rather, they indicate that the model should be interpreted as exploratory and hypothesis-generating until further external validation is available.
